# The Role of Adjuvant Chemotherapy in the Treatment of Esophageal Squamous Cell Carcinoma after Neoadjuvant Chemotherapy

**DOI:** 10.7150/jca.84484

**Published:** 2023-09-25

**Authors:** Keting Li, Wentao Hao, Xianben Liu, Yin Li, Haibo Sun, Shilei Liu, Wenqun Xing, Yan Zheng

**Affiliations:** 1 Department of Thoracic Surgery, The Affiliated Cancer Hospital of Zhengzhou University/Henan Cancer Hospital, Zhengzhou, China.; 2 Department of Thoracic Surgery, Cancer Institute and Hospital, Chinese Academy of Medical Sciences, BeiJing, China.

**Keywords:** esophageal squamous cell carcinoma, neoadjuvant chemotherapy, adjuvant chemotherapy, survival, prognosis.

## Abstract

**Background**: The aim of this study was to compare the efficacy of adjuvant chemotherapy after neoadjuvant chemotherapy in patients with esophageal squamous cell carcinoma (ESCC).

**Methods**: This retrospective study included patients diagnosed with ESCC at clinical stage T1N1-3M0 or T2-4N0-3M0. Six hundred and eleven patients underwent radical tumor surgical resection after neoadjuvant chemotherapy. Adjuvant chemotherapy was mainly a platinum-based combination regimen. Propensity score matching (PSM) was used to compare adjuvant chemotherapy (AC) vs. postoperative observation (POB) after surgery.

**Results**: A total of 611 patients were eligible, with 381 in the POB group and 230 in the AC group. POB group patients were younger (P=0.046) and at a later stage (ypT3/4: 127 [55%] vs. 177 [46%]), P=0.036; yPN+: 117[51%] vs. 3428[37%], P=0.001) before PSM. After 1:1 PSM, 213 pairs of patients were included in analysis. The 5-year overall survival (OS) was 60.6% and 57.2% in the POB and AC groups, respectively (HR 1.10, 95% CI: 0.80-1.51, P=0.562), and adjuvant chemotherapy did not improve OS compared with postoperative observation.

**Conclusions**: Postoperative adjuvant chemotherapy cannot improve the OS of patients with ESCC after neoadjuvant chemotherapy, but adjuvant chemotherapy tends to benefit ypN+ patients.

## Introduction

Esophageal carcinoma (EC) is one of the fastest-growing tumor types globally; it is the 9th most common malignancy and the 6th leading cause of cancer-related death in the world^1^. Neoadjuvant chemotherapy or neoadjuvant chemoradiation is the recommended treatment option for locally advanced, resectable esophageal cancer^2^-^5^; however, few data support the addition of adjuvant chemotherapy after neoadjuvant chemotherapy. Some retrospective studies have reached different conclusions; some believe that adjuvant chemotherapy after neoadjuvant therapy can improve overall survival (OS), whereas others do not. The potential benefit of adjuvant chemotherapy in patients receiving neoadjuvant therapy has not been studied in prospective trials. In retrospective studies, on the one hand, most of the pathological types studied were adenocarcinoma, and more than 90% of esophageal cancers in China are squamous cell carcinoma^6^; on the other hand, the neoadjuvant therapy strategies used were different, with neoadjuvant chemoradiotherapy being more common in Western countries and neoadjuvant chemotherapy being more common in China.

The aim of this study was to evaluate whether patients with esophageal squamous cell carcinoma who received preoperative chemotherapy benefitted postoperatively from the continuation of perioperative chemotherapy.

## Materials and Methods

### Patients

This retrospective study evaluated patients with primary esophageal squamous cell carcinoma who were treated at Zhengzhou University Affiliated Cancer Hospital (Henan Cancer Hospital) from January 1, 2015, to December 31, 2018. Patients were selected according to the following inclusion criteria: (1) clinical staging of T1N1-3M0 or T2-4N0-3M0, (2) neoadjuvant chemotherapy and radical esophagectomy, (3) postoperative pathologic confirmation of R0 resection, and (4) complete medical records. Patients were excluded according to the following criteria: (1) postoperative survival < 3 months, (2) R1/R2 resection, (3) postoperative adjuvant therapy other than chemotherapy and observation, and (4) incomplete postoperative pathological data. According to whether postoperative chemotherapy was performed, the patients were divided into an adjuvant chemotherapy (AC) group and a postoperative observation (POB) group.

All patients underwent transthoracic esophageal resection when the tumor was deemed resectable through this approach. Following resection, all tumor specimens were assessed by gastrointestinal pathology specialists. Data were collected retrospectively from case notes, and pathology reports were reviewed. All of the esophageal specimens were reclassified according to the Eighth Edition of the American Joint Committee on Cancer (AJCC8) staging manual into neoadjuvant pathological stage groups (ypTN).

### Surgical Procedure

At approximately 6-8 weeks after NAC, open (McKeown, left thoracic incision left cervical anastomosis) or MIE via thoracoscopy and/or laparoscopy was performed in the patients. Gastric tube reconstruction with a cervical anastomosis was performed to restore the continuity of the digestive tract. The range of lymphadenectomy included extensive mediastinal lymph node dissection. Bilateral laryngeal recurrent nerve lymph node dissection was requested for every patient. The abdominal nodes included the left gastric, para cardia, greater curvature, and lesser curvature. If the preoperative test showed that the resected neck lymph node had metastasized, then a 3-field lymph node dissection was needed.

### Follow-up

Patient death and recurrence data were collected during the follow-up process. To collect further information, patients were followed up by telephone or re-examination. The patients were followed up every 3 months in the first two years post-surgery, every 6 months for 3 to 5 years post-surgery, and every year after 5 years post-surgery. The latest follow-up evaluation was performed in January 2022. The follow-up time refers to the period from the time of operation to the patient's last follow-up visit. OS was defined as the time from radical surgery until the patient's death or last contact.

Recurrence events were assessed by the combination of postoperative CT scans, magnetic resonance imaging (MRI), and other clinical examinations. The patient's survival status and the results of related examinations were obtained by the researcher via inquiry about the previous follow-up and medical record data.

### Statistical Analysis

Continuous variables are described in terms of median and quartile ranges, and the Mann‒Whitney U test was used to compare the differences between two groups of continuous variables. Categorical variables were compared using a chi-square test. OS was defined as the time from surgery to death or the last follow-up. All variables were incorporated into the logistic regression curve to reduce selection and allocation bias between the two groups, and a propensity score was calculated. The match tolerance was set to 0.02, and a 1:1 matching was performed on the propensity score. The Kaplan‒Meier survival curve was plotted after matching to compare the OS of the two groups of patients. Univariate and multivariate survival analyses were performed using Cox proportional risk regression models to identify all factors that independently affect the survival of ESCC patients. Some continuous variables in the Cox proportional risk regression model were grouped by median as cutoff point. All statistical analyses were performed using SPSS 26, and P<0.05 was considered statistically significant.

## Results

### General Characteristics of the Patient Groups Before and After Matching

Between 2015 and 2018, thoracic surgeons performed a total of 2877 esophagectomy procedures, including minimally invasive esophagectomy (MIE) or open surgery (two-incision left thoracotomy or triple right thoracotomy). Among these 694 patients receiving surgery after neoadjuvant chemotherapy, a total of 611 were analyzed after exclusion. There were 381 cases (62%) in the POB group and 230 (38%) in the AC group. Patients treated with AC may be younger than patients in the POB group (61 years vs. 63 years; P<0.05), had a later stage (ypT3/4:127[55%] vs.177[46%]; P<0.05; ypN+:117[51%] vs. 3428[37%]; P<0.05), and fewer lymph nodes dismissed (24 vs. 27; P<0.05).

**Figure 1:**
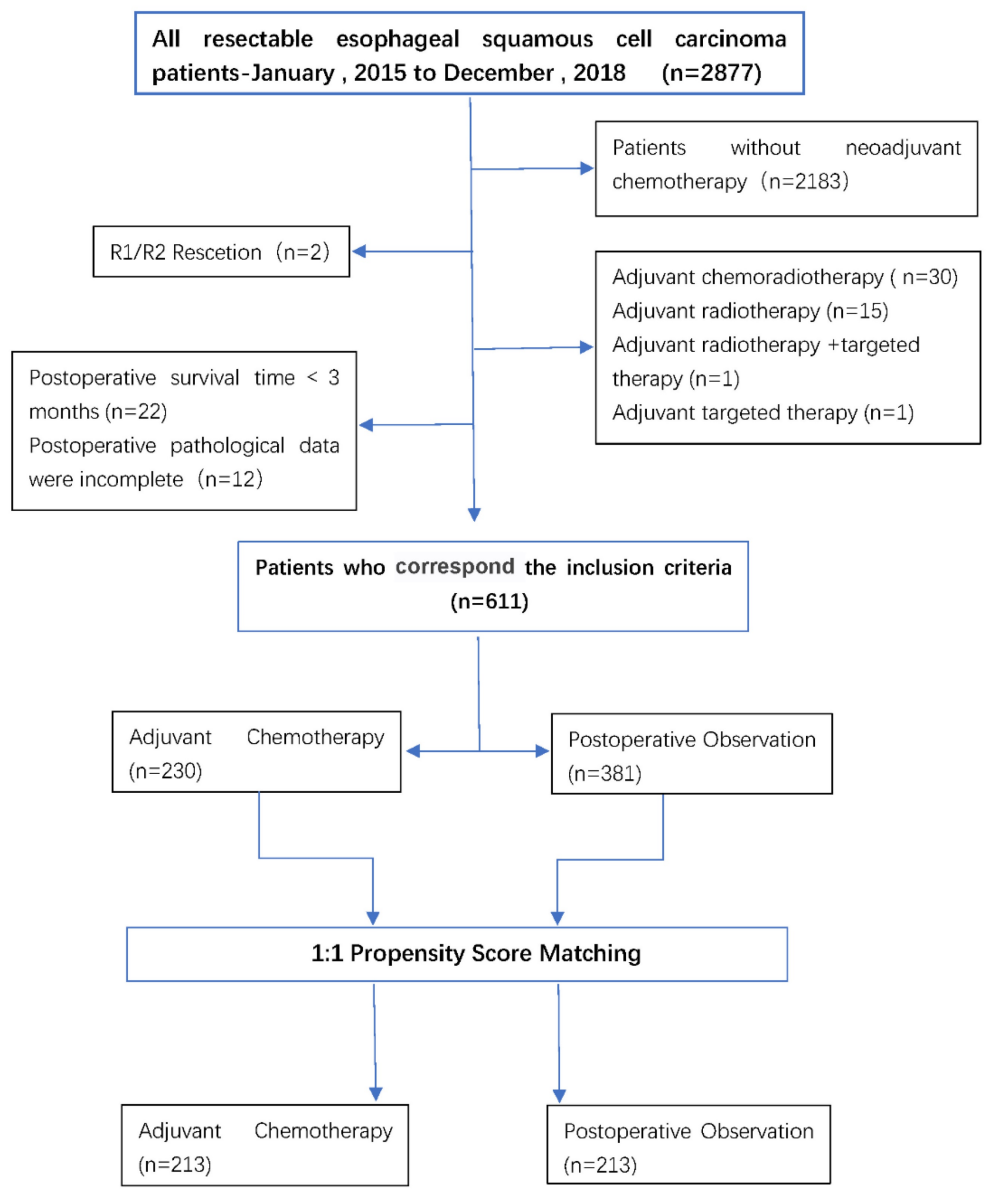
A CONSORT-style flow diagram of participant disposition (whole study cohort).

A 1:1 match was made based on the propensity score, with 213 patients in the AC group being matched to 213 patients in the POB group, with a match rate of 93%. In the matching data, the baseline covariates between the two treatment groups were fully matched, including age, sex, degree of tumor differentiation, cT stage, cN status, ypT stage, ypN stage, the number of cleaned lymph nodes, the number of positive lymph nodes, surgical methods, etc. Table 1 summarizes the selected baseline characteristics in the postmatch data before and after matching for each patient in both treatment groups. The patient flow for the study is shown in Figure 1.

### Comparisons of Survival After Propensity Score Matching

**Figure 2:**
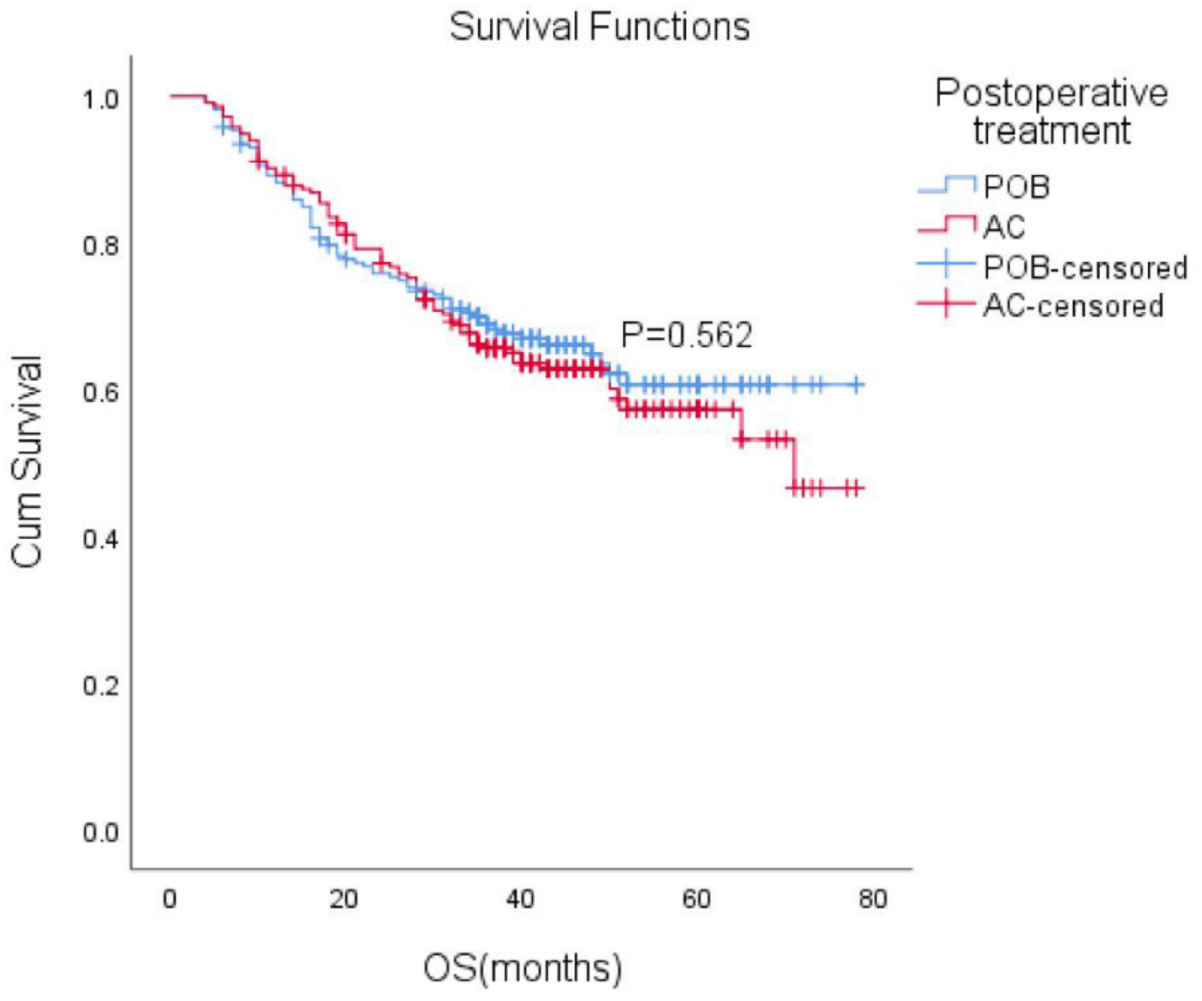
Overall survival (OS) with or without postoperative chemotherapy in patients who received neoadjuvant therapy (P = 0.562).

After matching, the median follow-up time for the entire paired cohort was 38 months (IQR, 23-48 months), and among those patients who achieved a complete response (CR) of 7.2% (44/611), the median OS was not reached. Furthermore, the median OS was not reached in either group. The estimated overall survival rates at 1, 3, and 5 years were 88%, 66%, and 61% in the POB group and 89%, 66%, and 57% in the AC group, respectively. Adjuvant chemotherapy in the AC group did not improve OS compared with the POB group (HR, 1.10, 95% CI, 0.80-1.51, P=0.562 Figure 2).

In the K-M analysis, six variables, including sex, degree of differentiation, cN status, ypN stage, ypT stage and surgical method, affected postoperative survival. The variables with P<0.1 were included in the Cox multivariate analysis, and cN status, ypN stage, ypT stage and surgical methods affected prognosis and long-term survival (Table 2).

**Figure 3:**
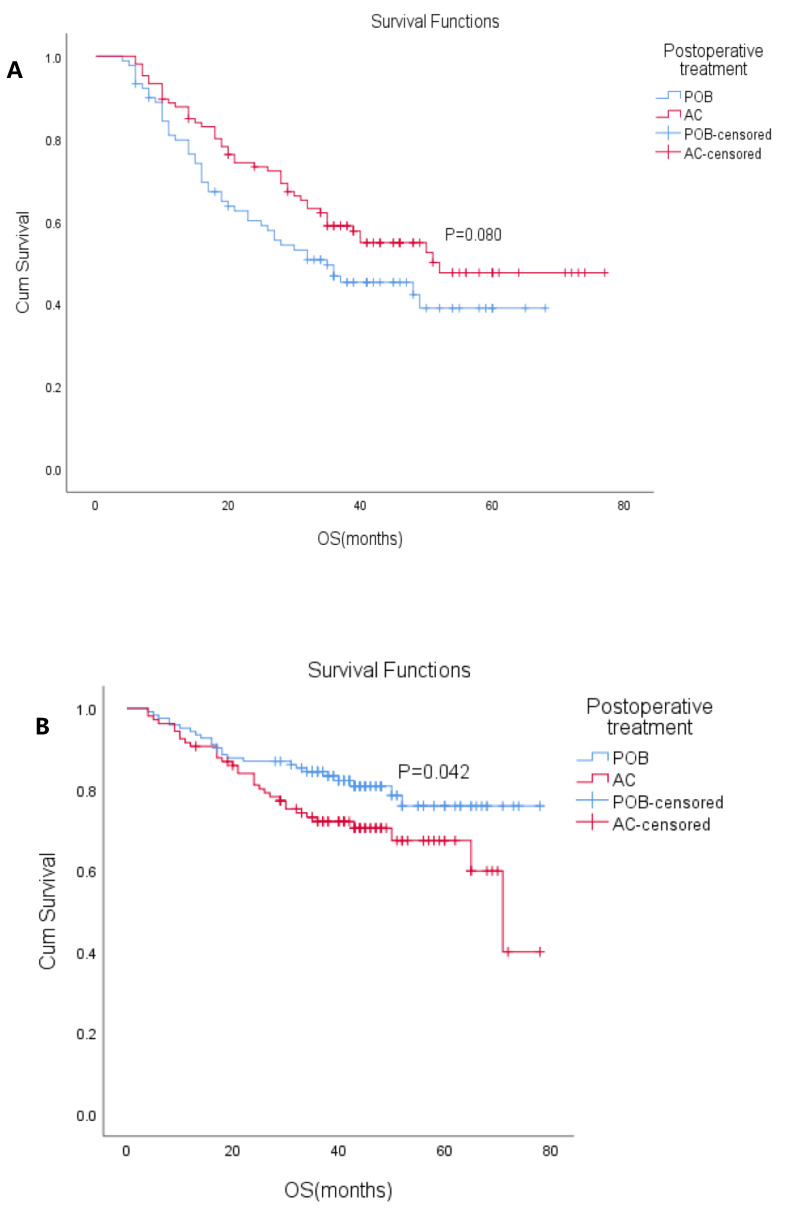
** (A)** Overall survival (OS) with or without postoperative chemotherapy in ypN+ patients who received neoadjuvant therapy (P=0.080). **(B)** Overall survival (OS) with or without postoperative chemotherapy in ypN- patients who received neoadjuvant therapy. (P=0.042)

The patients were divided into ypN (+) and ypN(-) subgroups according to ypN status (positive/negative) (Table 3). In the ypN(+) subgroup, the median OS was 48 months (95% CI: 37.7-58.3), the median OS in the POB group was 35 months (95% CI: 18.2-51.8), and the median OS was not reached in the AC group. The 5-year OS in the POB and AC groups was 39% and 48%, respectively (HR 0.704, 95% CI: 0.47-1.05, P=0.08). K-M analysis showed that cN status, ypT stage, and surgical modalities affected survival. Postoperative adjuvant chemotherapy did not result in OS changes in patients within the ypN(+) subgroup, but there was a trend of benefit (P=0.08 Figure 3A). In the ypN(-) subgroup, the median OS was not reached, the median OS was not reached in the POB group, and the median OS was 71 months (95% CI: 60.8-81.2) in the AC group. The 5-year OS in the POB and AC groups was 75.9% and 67.4%, respectively (HR 1.71, 95% CI: 1.01-2.90, P=0.042 Figure 3B), and the 5-year survival rate after adjuvant chemotherapy was reduced. Univariate analysis showed that adjuvant chemotherapy, cT stage, ypT stage, degree of differentiation and surgical methods affected survival. When the variables with a P<0.1 were included in the multivariable analysis, only surgical method was found to affect survival.

## Discussion

Previous studies have shown that preoperative adjuvant therapy can improve the prognosis of patients^2^-^5^, and that the combination of surgery and postoperative adjuvant chemotherapy can better prevent recurrence and metastasis than surgery alone, especially for patients with postoperatively confirmed pathological lymph node positivity^7^. However, there was no difference in OS in our study. Preoperative chemotherapy improves patient survival benefits compared with postoperative chemotherapy, yet there are few studies on the use of adjuvant chemotherapy after preoperative chemotherapy, and no firm conclusions have been drawn as to whether this regimen benefits patients. In our study, AC did not improve patient OS compared to POB, and the same conclusion was reached in subgroup analyses, but in ypN+ patients receiving AC there was a trend of benefit (P=0.08).

The rationale for adjuvant chemotherapy to improve survival is to reduce the rate of distant recurrence. Long-term follow-up results from some studies suggests that a high proportion of patients receiving neoadjuvant therapy still have disease progression, affecting long-term survival after surgery^5^,^8^-^10^. In our study, less than 40% (230/694) of patients received adjuvant chemotherapy postoperatively. Patients who received adjuvant chemotherapy were younger (61 years versus 63 years; P<0.05), had an advanced pathologic stage (ypT3/4:127[55%] vs. 177[46%]; P<0.05; ypN+:117[51%] vs. 3428[37%]; P<0.05), and had fewer lymph nodes removed (24 versus 27; P<0.05).

Kim et al. 11 demonstrated that adjuvant chemotherapy after neoadjuvant chemoradiotherapy is feasible; however, the small sample size (n=145) may have affected the results of the study. The Mokda et al.^12^ study reached the same conclusion, including up to 10,000 patients who received neoadjuvant chemoradiation preoperatively, but the number of postoperative adjuvant chemotherapies was small (n = 814). In addition, there was a lack of bulk sample data analysis, and no detailed chemotherapy regimen was documented. Studies by T. Glatz et al.^13^ showed that adjuvant chemotherapy given after neoadjuvant chemotherapy can improve OS. Another esophageal adenocarcinoma study^14^ reached the same conclusion, with patients who had received neoadjuvant therapy followed by adjuvant chemotherapy showing an improved overall survival, even in patients with postoperative pathologic evidence of node-negative or margin-negative status; in addition, a more pronounced survival benefit was noted in patients whose response to neoadjuvant therapy was stable or degraded, which could be understood to be the result of a sensitivity to chemotherapy drugs. In these studies, the main pathological type of esophageal cancer investigated was adenocarcinoma, and the main neoadjuvant strategy used was chemoradiotherapy; so these treatment strategies can only be used for reference.

A study of esophageal squamous cell carcinoma showed that adjuvant therapy after neoadjuvant therapy improved disease-free survival (DFS) without a pathological response, and there was no difference in OS. The reason for the lack of OS difference may be that there were more deaths from nonneoplastic causes in patients in the adjuvant therapy group. In this study, chemoradiation was used preoperatively and postoperatively^7^.

However, there are also studies that offer different conclusions. A multicenter cohort study^15^ showed that adjuvant chemotherapy given after neoadjuvant chemotherapy for esophageal adenocarcinoma did not improve prognosis, and only benefitted patients with R1 resection. A study by Rebecca K. Bott et al. [16]reached a similar conclusion. The findings of these two studies are similar to our results.

When univariate and multivariate analyses of the matched data were performed, it was found that cN status, ypN status, ypT stage and surgical methods may affect the prognosis, which is in line with expectations, as cN+, ypN+, and ypT3-4 indicates that the disease is further in its progression and that the prognosis may be worse. The surgical approach also affects prognosis, and in our study OS with MIE was preferred, unlike previous studies^17^. This may be explained by the fact that these procedures are performed by different surgeons and that the doctors who perform the MIE procedure are more experienced,and some open thorax patients are due to intraoperative hemorrhage during MIE or severe adhesion of tumor tissues to adjacent tissues, which leads to intermediate open thorax, as well as the advantages of MIE in lymph node dissection compared with open thoracic surgery, which are factors that may affect the survival of patients with open thoracic surgery.

Justin Drake et al. 18 concluded that ypN+ patients receiving neoadjuvant chemotherapy followed by adjuvant chemotherapy postoperatively improves OS, and this study only included ypN+ patients. The study by Burt et al. 19 came to the same conclusion. In our ypN(+) subgroup analysis, adjuvant chemotherapy, although not prognostic, showed a trend of benefit (P=0.08 and P=0.053) and although univariate and multivariate analysis did not reveal adjuvant chemotherapy to be an independent prognostic factor for survival, this result suggests that screened patients may benefit from adjuvant chemotherapy if patients are adequately assessed postoperatively. In the ypN(-) subgroup analysis, the 5-year OS in the AC group was statistically lower than that found in the POB group (P=0.042). This can be explained by patients who have experienced the challenges of neoadjuvant chemotherapy and surgery, and the postoperative score from the Karnofsky Performance Scale (KPS) is often low and tolerance for postoperative adjuvant therapy is limited. This result partly reflects the fact that postoperative adjuvant therapy may not be necessary in this subset of patients.

The pathological response profile after neoadjuvant therapy is also a matter of concern; pathological response is defined by the standard tumor regression grade (TRG), which is a well-recognized prognostic factor^20^. The relationship between different pathological responses and adjuvant therapy efficacy is also a hotly debated issue, and the pathological response profile after neoadjuvant therapy may also inform the selection of postoperative adjuvant regimens, which was not in our study assess the pathological response of patients after neoadjuvant therapy, a European study of 134 patients undergoing NAT, i.e., adjuvant chemotherapy has a survival benefit in patients with low-stage or no tumor response^21^. In contrast, a single-institution study in the United Kingdom demonstrated a significant increase in survival in patients with chemotherapy-responsive tumors (Mandard 1-3) who received AC chemotherapy (the MAGIC regimen), but no significant improvement in patients with nonresponsive tumors (Mandard 4-5) 22. The role of adjuvant chemotherapy in the subgroups of non or low responders needs to be explored in subsequent studies.

Our study has certain limitations, and ideally, a randomized prospective clinical trial would be a better study design to address whether adjuvant chemotherapy actually benefits patients who have received neoadjuvant chemotherapy. In fact, due to the lack of evidence from such clinical trials to date, we sought evidence by analyzing previous cases through retrospective analysis. Despite using relatively large cohort, the study design did not allow us to completely eliminate selection bias, although the propensity matching did balance confounding factors between the two groups. All of the studies utilized were retrospective, nonrandomized, and single-center, and the decision for patients to receive adjuvant therapy was largely determined by clinicians, with some selectivity. Due to the fact that postoperative adjuvant chemotherapy cases are rare, bulk sample analysis was lacking. Second, less than 80% of all patients completed 2 cycles of neoadjuvant chemotherapy, and only 62% completed 2 cycles of adjuvant chemotherapy. Factors such as insufficient chemotherapy intensity may affect long-term survival after surgery. Finally, we used a wide range of chemotherapy regimens, including but not limited to the following: paclitaxel/docetaxel + platinum, S-1, 5-FU + platinum. Some other patients (10%) received neoadjuvant chemotherapy in an external hospital, and the specific regimen is unknown. We did not include information about chemotherapy toxicity or postoperative complications in our study, and some serious adverse effects may have affected subsequent treatment. Due to the limitations of the retrospective study, we did not collect the pathological responses of the patients after receiving neoadjuvant therapy. The decision as to whether a patient needs adjuvant chemotherapy after surgery and the regimen of chemotherapy is made by the physician at that time based on the relevant examination results of the patient and the patient's own tolerance.

Overall, adjuvant chemotherapy after neoadjuvant chemotherapy did not improve OS compared with postoperative observation in our study. Given the differing conclusions from multiple studies about the benefits of adjuvant therapy after neoadjuvant chemotherapy, randomized clinical trials are needed.

## Figures and Tables

**Table 1:** Comparison of Select Baseline Variables Between the Postoperative Observation (POB) and Adjuvant Chemotherapy (AC) Groups in the Original (Unmatched) and the Matched Data Sets.

Variable	Original (Unmatched) Data			Matched Data		
	No. (%)		PValue	No. (%)		P Value
	POB n=381	AC n=230		POB n=213	AC n=213	
Age median	63(57-68)	61(55-67)	0.046	60(55-66)	61(55-67)	0.404
gender			0.659			0.914
male	272	168		154	155	
female	109	62		59	58	
cT			0.010			0.976
1	5	0		1	0	
2	88	41		37	41	
3	280	176		169	163	
4	8	13		6	9	
cN			0.247			0.923
+	159	107		101	100	
-	222	123		112	113	
ypT			0.019			0.078
0	37	14		33	14	
1	76	40		42	39	
2	91	49		31	47	
3	173	121		104	107	
4	4	6		3	6	
ypN			0.001			0.233
0	239	113		123	107	
1	91	70		54	67	
2	32	43		23	35	
3	19	4		13	4	
Tumor differentiation			0.269			0.319
Well	17	5		5	5	
moderate	132	76		61	71	
Poor	232	149		147	137	
Surgical approach						0.591
MIE	311	158		155	150	
thoracotomy	70	72		58	63	
Lymph node yield Median (IQR)	27(21-35)	24(19-32)	0.001	24(20-31)	24(19-32)	0.960
Positive lymph nodes Median (IQR)	0(0-1)	1(0-2)	0.001	0(0-2)	0(0-2)	0.144

**Table 2:** Univariate and multivariate Cox analysis for esophageal cancer-specific survival rates.

Variable	After matching			
	Univariate analysis		Multivariable analysis	
	HR (95% CI)	P^a^	HR (95% CI)	P^b^
Postoperative adjuvant chemotherapy (ref: POB)				
AC	1.098(0.800-1.507)	0.562		
Age, years (ref: <60)				
≥60	1.055(0.763-1.459)	0.746		
Sex (ref: Male)				
Female	0.645(0.439-0.948)	0.026	0.750(0.507-1.109)	0.149
Differentiation (ref: Well- moderate)				
Poor	0.646(0.467-0.894)	0.008	0.771(0.547-1.086)	0.136
cT (ref: T1-2)				
T3-4	1.271(0.828-1.950)	0.273		
cN (ref:-)				
+	2.075(1.501-2.869)	<0.001	1.625(1.140-2.317)	0.007
ypT (ref: T0-2)				
T3-4	2.449(1.741-3.446)	<0.001	1.896(1.317-2.729)	<0.001
ypN (ref:-)				
+	2.442(1.759-3.390)	<0.001	1.844(1.276-2.664)	<0.001
Lymph node yield (ref: <24)				
≥24	0.894(0.651-1.227)	0.488		
Surgical approach(ref:MIE)				
Thoracotomy	2.155(1.561-2.976)	<0.001	2.264(1.140-2.317)	<0.001

^a^Univariate analysis by KaplaneMeier method with log-rank test for the comparison of subgroups. ^b^Multivariate survival analysis by the Cox proportional hazard model (forward selection strategy using a likelihood ratio statistic) including the report of relative risks and their 95%-confidential intervals.

**Table 3:** Analysis of esophageal cancer-specific survival rates stratified by ypN(+/-).

Variable	After matching			
	ypN-		ypN+	
	HR (95% CI)	P	HR (95% CI)	P
Postoperative adjuvant chemotherapy (ref: POB)				
			
AC	1.713(1.012-2.899)	0.045	0.704(0.473-1.049)	0.085
Age, years (ref: <60)				
≥60	0.924(0.544-1.570)	0.771	1.207(0.801-1.820)	0.368
Sex (ref: Male)				
Female	0.736(0.408-1.328)	0.308	0.665(0.398-1.109)	0.118
differentiation(ref: Well- moderate)				
Poor	0.562(0.330-0.956)	0.033	0.707(0.468-1.066)	0.098
cT(ref: T1-2)				
T3-4	2.822(1.021-7.800)	0.045	1.048(0.646-1.699)	0.850
cN(ref:-)				
+	1.527(0.885-2.635)	0.128	1.622(1.022-2.576)	0.040
ypT(ref: T0-2)				
T3-4	2.441(1.431-4.165)	0.001	1.944(1.240-3.048)	0.004
Lymph node yield(ref:<24)				
≥24	0.668(0.393-1.135)	0.136	0.919(0.611-1.380)	0.682
Surgical approach(ref:MIE)				
Thoracotomy	2.356(1.395-3.980)	0.001	2.219(1.469-3.351)	<0.001

## Abbreviations

ESCC: esophageal squamous cell carcinoma
PSM: Propensity score matching
AC: adjuvant chemotherapy
POB: postoperative observation
EC: Esophageal carcinoma
OS: overall survival
MIE: minimally invasive esophagectomy
DFS: disease-free survival
TRG: tumor regression grade
KPS: Karnofsky Performance Scale
